# Infliximab-linked gut microbiome signatures as candidate treatment response biomarkers in pediatric inflammatory bowel disease: a systematic review

**DOI:** 10.3389/fphar.2026.1877033

**Published:** 2026-07-10

**Authors:** Hala Altaher, Mustafa Al-Mashhadani, Rameen Usman, Hardik Ghelani, Reem K. Jan

**Affiliations:** College of Medicine (CoM), Mohammed Bin Rashid University of Medicine and Health Sciences (MBRU), Dubai Health, Dubai, United Arab Emirates

**Keywords:** anti-TNF, dysbiosis, inflammatory bowel disease, infliximab, microbiome, pediatric, systematic review

## Abstract

**Background:**

Infliximab (IFX) is a chimeric monoclonal antibody against tumor necrosis factor-alpha (TNF-α) that is widely used for induction and maintenance therapy in pediatric inflammatory bowel disease (IBD), yet its effects on the developing intestinal microbiome and treatment response remain unclear.

**Objective:**

To systematically synthesize evidence on IFX-associated microbiome changes in pediatric IBD and evaluate microbiome features linked to treatment response.

**Methods:**

A systematic review was conducted following the PRISMA 2020 guidelines. PubMed, Scopus, Embase, and CENTRAL were searched from database inception until March 2026, and our results were synthesized narratively.

**Results:**

Of the 945 records identified, 13 studies met the inclusion criteria, comprising 242 pediatric patients. Findings for alpha diversity were variable across studies. In contrast, beta-diversity patterns, taxonomic profiles, and functional analyses more consistently showed positive IFX-associated microbial changes. Responders to treatment more frequently showed enrichment of beneficial taxa, including *Faecalibacterium, Subdoligranulum,* and *Bifidobacterium*, while non-responders exhibited a tendency towards dysbiotic microbial signatures, evidenced by increased levels of *Gammaproteobacteria* and *Candida*. Functional and metabolomic studies suggested beneficial shifts in bile-acid metabolism, short-chain fatty acid-related pathways, and inflammatory signaling post IFX treatment. Clinical and biochemical improvements with IFX were consistently reported; however, these improvements were not always accompanied by uniform recovery of overall microbial diversity.

**Conclusion:**

In pediatric IBD, IFX is more consistently associated with shifts in microbial composition and function than with broad increases in overall microbial diversity. Further large, standardized studies are warranted to refine the role of microbiome features in biologic treatment stratification.

**Systematic Review Registration:**

https://www.crd.york.ac.uk/PROSPERO/view/CRD420251133625, identifier CRD420251133625.

## Graphical Abstract

**Figure:**
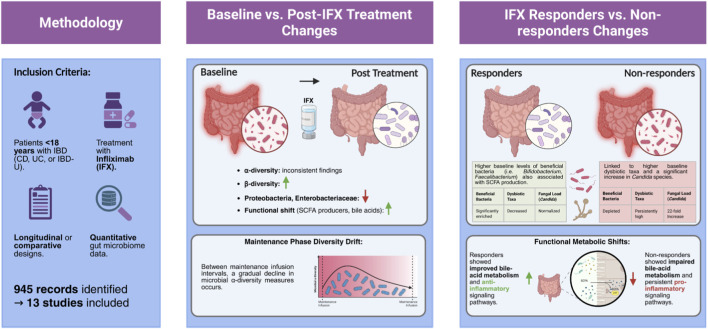
Infliximab-linked gut microbiome signatures as treatment-response biomarkers in pediatric inflammatory bowel disease: a systematic review.

## 1 Introduction

Inflammatory bowel disease (IBD) is a chronic and relapsing inflammatory condition of the gastrointestinal tract attributed to immune dysregulation and can be divided into Crohn’s disease (CD) and ulcerative colitis (UC) (Mah et al., 2023; McDowell et al., 2025). Pediatric-onset IBD often presents with a more aggressive and extensive disease phenotype compared with adult-onset disease, making it more challenging to manage. As a result, there is a greater need for earlier escalation to advanced therapies, such as biological agents at younger ages (Moon, 2019; Krauthammer et al., 2023). Biological therapies have transformed the management of IBD by targeting key immune pathways involved in chronic intestinal inflammation, enabling improved disease control and mucosal healing in patients with moderate-to-severe disease. One of these biological agents is infliximab (IFX), a monoclonal antibody against tumor necrosis factor-α (TNF-α), which is widely used in pediatric practice for both induction and maintenance of IBD remission (Fatima et al., 2025; Parashette et al., 2010).

The gut microbiome refers to the community of microorganisms inhabiting the gastrointestinal tract and their collective genetic material, and it plays critical roles in host homeostasis and health outcomes (Thursby and Juge, 2017), impacting immunological functions, and inflammation (Qiu et al., 2022; Shan et al., 2022). Dysbiosis (which occurs when the normal and healthy gut microbiome is shifted to a dysfunctional assortment of organisms) has been associated with IBD pathogenesis and disease activity; therefore, the microbiome is an important consideration in IBD research and management (Shan et al., 2022; Tyszkowski and Mehrzad, 2023; Santana et al., 2022; Dahal et al., 2023; Hetta et al., 2024). In pediatric IBD, the microbiome is disrupted, indicated by reduced diversity of organisms and compositional shifts (Fitzgerald et al., 2021; Iliev et al., 2025; Maeder and Nguyen, 2025). Studies have demonstrated changes in the microbiome following IFX infusion, impacting the diversity and the composition of the microbiome (Seong et al., 2020; Carlsen et al., 2024). Some studies suggest that IFX may shift the pediatric microbiome toward a healthier profile, whereas others report no significant change or only short-term diversity changes (Seong et al., 2020; Carlsen et al., 2024; Kowalska-Duplaga et al., 2020). Because the pediatric microbiome is still developing, microbiome alterations associated with IFX may differ from those observed in adults and may have distinct implications for disease course and treatment response (Seong et al., 2020; Shin et al., 2019; Gaufin et al., 2018).

Despite its established role in IBD, response to IFX is not uniform. Primary non-response during induction and secondary loss of response after initial benefit remain important clinical limitations, and long-term prospective data indicate that sustained remission is not achieved in a substantial proportion of anti-TNF-treated patients (Chanchlani et al., 2024; Peyrin-Biroulet et al., 2021; Ding et al., 2016). As a result, there is growing interest in exploratory biomarkers that may help predict, stratify, or monitor therapeutic response earlier and more reliably than clinical assessment alone (Marsal et al., 2022). The gut microbiome is a potential candidate because it is closely associated with intestinal inflammation, and because response-associated signatures appear closely involve specific microbial taxa and metabolic functions rather than broad diversity measures alone (Durack and Lynch, 2019; Al et al., 2020; Teofani et al., 2022). Recent evidence suggests associations between baseline microbiome characteristics and post-treatment microbiome disruption may impact the pharmacokinetics of IFX and alter treatment responses (Höyhtyä et al., 2023; Radhakrishnan et al., 2022). Understanding these relationships in pediatric IBD may help identify exploratory microbiome-based markers for future monitoring or prognosis, but these markers require validation before routine clinical use. While published systematic reviews have examined the microbiome in pediatric IBD or the impact of various therapies in mixed-age cohorts, no prior registered or published review has specifically synthesized evidence on IFX-induced microbiome changes and their potential influence on treatment response in pediatric populations, highlighting a clear gap in the literature that this review aims to address.

## 2 Methodology

This systematic review was conducted and reported in accordance with the PRISMA 2020 statement (see Supplementary Material S1 for the filled-in PRISMA checklist) (Page et al., 2021). It was prospectively designed with a protocol adhering to Preferred reporting items for systematic review and meta-analysis protocols (PRISMA-P) guidelines (Shamseer et al., 2015). The full protocol can be found publicly available on PROSPERO (PROSPERO registration code: CRD420251133625) with the only deviation being a correction of the original protocol due to a spelling mistake made in one of the search terms which was accordingly reported on PROSPERO with publicly accessible time-stamps and justification.

### 2.1 Review objectives

This systematic review was guided by a Population, Intervention, Comparator, Outcome, and Study design (PICOS) framework (Frandsen et al., 2020) as shown in Box 1. This review aimed to systematically evaluate the impact of IFX therapy on the gut microbiome in pediatric patients (<18 years of age, or pediatric as defined by the original study authors) with IBD and to assess whether microbiome features were associated with treatment response or outcomes. The primary objectives were to summarize within-patient changes in gut microbiome composition, diversity, and function following IFX therapy and to determine whether baseline or early-treatment microbiome features can be associated with IFX response. Secondary objectives were to compare microbiome differences between responders and non-responders and evaluate the influence of potential concomitant medications on reported patient outcomes. To assist with consistency in evaluating the review objectives and to help readers with the contents of this review, we defined and set certain key terms with definitions as reported in Supplementary Material S3 that encompassed clinical and microbiome-specific terminology (Shan et al., 2022; Levine et al., 2014; Papamichael et al., 2019; Aardoom et al., 2019; Hyams et al., 2007; Petersen and Round, 2014; Jovel et al., 2016; Callahan et al., 2017; Bustin et al., 2009; Ruemmele et al., 2015; Koninckx et al., 2021; Upadhyay et al., 2023).

BOX 1 **The PICOS-based framework used for inclusion/exclusion of studies during the title-and-abstract and full-text screening.**

Population

Does the study involve human pediatric participants aged <18 years (or pediatric as defined by the authors) with a confirmed diagnosis of inflammatory bowel disease (e.g., Crohn’s disease, ulcerative colitis, or IBD-unclassified)? *YES/NO*.

If *NO* exclude.

In mixed-age populations, can pediatric-specific data be extracted separately? *YES/NO*.

If *NO* exclude.

Intervention

Does the study evaluate infliximab treatment or exposure? *YES/NO*.

If *NO* exclude.

Comparator/context

Does the study include a relevant comparator or analytical context (e.g., pre-vs post-IFX, baseline vs treatment, responders vs. non-responders, or IFX-specific outcomes reported separately from other therapies)? *YES/NO*.

If *NO* exclude.

Outcome

Does the study report gut microbiome outcomes (e.g., alpha diversity, beta diversity/community composition, taxonomic abundance, gene pathways, or functional/metabolomic findings)? *YES/NO*.

If *NO* exclude.

Does the study report treatment response outcomes to IFX or microbiome features associated with treatment response? *YES/NO*.

If *NO* exclude.

Study design

Is this study an empirical (evidence-based) and/or original human research article? *YES/NO*.

If *NO* exclude.

### 2.2 Literature search

This systematic review searched four major electronic databases (PubMed, Scopus, Embase, and Cochrane CENTRAL) from database inception until 2 March 2026. The search strategy was initially drafted with relevant Medical Subject Headings [Mesh] terms and keywords according to the review PICOS framework plotted in table format to highlight relevant keywords and MeSH terms (see Supplementary Material S2). To account for database indexing and search mechanism differences, database-specific search strategies were tailored to optimize sensitivity and specificity. These search strategies involved PICOS-based search strings utilizing Boolean (AND/OR) operators optimized in a manner that yielded the highest number of search results per respective database. No language restrictions were applied. The verbatim search strategies were prospectively published, which may be found publicly available in the original PROSPERO registration and as follows: PubMed (“Inflammatory Bowel Diseases” [Mesh] OR “Crohn Disease” [Mesh] OR “Colitis, Ulcerative” [Mesh] OR “inflammatory bowel disease” [tiab] OR “Crohn*” [tiab] OR “ulcerative colitis” [tiab] OR IBD [tiab]) AND (“Infliximab” [Mesh] OR infliximab [tiab] OR Remicade [tiab] OR “Tumor Necrosis Factor-alpha” [Mesh] OR “Tumor Necrosis Factor Inhibitors” [Mesh] OR “anti-TNF” [tiab] OR “TNF inhibitor*” [tiab]) AND (“Gastrointestinal Microbiome” [Mesh] OR “Microbiota” [Mesh] OR microbiome [tiab] OR microbiota [tiab] OR dysbiosis [tiab] OR “gut flora” [tiab] OR metagenomic*[tiab] OR “16 S” [tiab] OR “shotgun sequencing” [tiab] OR “alpha diversity” OR “beta diversity”); Scopus (TITLE-ABS-KEY (“inflammatory bowel disease” OR “Crohn*” OR “ulcerative colitis” OR IBD)) AND (TITLE-ABS-KEY (infliximab OR remicade OR “anti-TNF” OR “TNF inhibitor*” OR “tumor necrosis factor inhibitor*”)) AND (TITLE-ABS-KEY (microbiome OR microbiota OR dysbiosis OR “gut flora” OR metagenomic* OR “16S” OR “shotgun sequencing” OR “alpha diversity” OR “beta diversity”)) AND (TITLE-ABS-KEY (pediatric OR paediatric OR child* OR adolescen* OR teen* OR youth)); Embase (‘inflammatory bowel disease’/exp OR ‘inflammatory bowel disease’) AND ‘infliximab’ AND ‘microbiome’:ti,ab,kw; Cochrane CENTRAL (“infliximab”):ti,ab,kw AND (“microbiome”):ti,ab,kw AND (“pediatric”):ti,ab,kw.

### 2.3 Study selection process

The study selection process was conducted using the Covidence software (WCM, 2020), which served as a platform for systematic review management. The Covidence platform was used for title-and-abstract screening, full-text review, reviewer blinding, duplicate removal, PRISMA flow diagram generation, and adherence to PRISMA 2020 guidelines (Page et al., 2021). Duplicate records were removed automatically by the Covidence software and manually, where necessary.

A predefined PICOS-based eligibility rubric (see Box 1) was designed by and available to all reviewers (MA, HA, RU) during title-and-abstract and full-text screening to serve as a standard checklist for including/excluding studies. Study selection was conducted in two stages: title-and-abstract screening followed by full-text review. At each stage, articles were independently assessed by two reviewers (MA, HA, RU), each blinded to the other reviewers’ decisions. Articles progressed to the next stage if they received two “Yes” votes or one “Yes” and one “Maybe” vote. Articles receiving two “No” votes were excluded. Any other voting combination was placed into a conflict section where a third independent reviewer (MA, HA, or RU) who had not previously voted on the article made a final blinded decision.

### 2.4 Data extraction

After full-text screening, all included studies underwent data extraction via a standardized Microsoft Excel form developed from the review protocol and reported verbatim as per Box 2. Data extraction was performed independently by at least two reviewers (HA, RU, MA) per article and its supplementary material. A consensus extraction was then generated for each study, with disagreements resolved through discussion or adjudication by a third reviewer (MA, HA, or RU). Because the included studies varied in methodology and reporting, some did not report complete quantitative details for all outcomes, so unreported data was documented transparently in our extraction as “Not Reported”. Extracted data included study identification details, publication year, country, setting, study design, pediatric sample size, demographic characteristics, IBD subtype, disease duration or severity, IFX regimen, timing of sampling, comparator type, microbiome sample type, sequencing or analytical method, alpha diversity measures, beta diversity measures, taxonomic findings, functional or metabolomic findings, longitudinal microbiome changes, clinical response or remission outcomes, biochemical and endoscopic outcomes where available, microbiome features linked to treatment response, and key statistical measures. Funding sources, conflicts of interest, and author-reported limitations were also recorded. Furthermore, potential cohort overlap across publications was assessed during full-text review and data extraction using author groups, study setting, recruitment characteristics, sample size, treatment phase, and reported participant characteristics. Because reporting was insufficient to confirm independence in all cases, potentially overlapping publications were retained as they contributed non-identical outcomes or follow-up information, and this was considered during the interpretation of evidence.

BOX 2 **The standardized data extraction form used for all included studies (n = 13) to collect study characteristics, infliximab-related variables, microbiome and clinical outcomes from each included study. Missing or unreported data was recorded as such.**

Study identification

Digital Object Identifier (DOI)

Author(s)

Study title.

Journal.

Year of publication.

Country.

Setting.

Reviewer notes/extraction notes.

Methods

Study design.

Bioinformatic pipeline.

Population

Pediatric sample size.

Sex distribution.

IBD subtypes.

Disease duration/severity.

Age range (years)

Mean age (years) ± SD.

Median age (years)

Number with Crohn’s disease

Number with ulcerative colitis.

Number with unclassified IBD.

Intervention (IFX)

Number of participants allocated.

IFX dose.

Infusion schedule.

IFX brand.

Concomitant medications.

Sampling timing.

Treatment phase (induction, maintenance, or withdrawal)

Prior therapies.

Outcomes

Sample type(s)

Number of analyzed samples.

Sequencing method.

Alpha diversity.

Beta diversity.

Taxonomic findings.

Functional/metabolic findings.

Author-reported longitudinal changes.

Clinical response/remission.

Biochemical response.

Endoscopic/histological response.

Other outcomes.

### 2.5 Data synthesis

We used a descriptive narrative synthesis to report our findings and to examine the effects of IFX on the gut microbiome in pediatric IBD. A meta-analysis was initially planned where (i) reported outcomes were comparable, (ii) methodological approaches of included studies could provide a reliable effect direction via a meta-analysis, and (iii) sufficient statistical data were available for pooling; however, following data extraction, quantitative pooling was not appropriate due to variation in study design and reporting. Instead, a structured narrative synthesis was undertaken to identify consistent patterns across studies. Variation across studies reflected differences in study populations, comparator groups, microbiome sampling timepoints, sequencing approaches, and outcome reporting, which were systematically examined during synthesis. Particularly, microbiome diversity outcomes were reported using different metrics across studies, with different alpha and beta diversity indices and limited compatible numerical data, which limited formal pooling but allowed identification of consistent directional trends across studies. Therefore, we synthesized and reported our findings narratively to identify patterns, consistencies, divergences, and methodological gaps across the evidence base. Additionally, graphical representation of the abstract (Graphical abstract) was conducted using BioRender (https://www.biorender.com; Science Suite Inc.; Toronto, Canada; accessed 15 March 2026) to aid in summarizing our findings.

### 2.6 Quality assessment

All studies included in this systematic review were critically appraised via the Risk of Bias in Non-randomized Studies of Interventions (ROBINS-I) tool due to the observational nature of the included studies evaluating the IFX intervention (Sterne et al., 2016). No randomized-control studies were found eligible for inclusion, so no other tools were used for risk-of-bias assessment. ROBINS-I enabled appraisal across key domains including bias due to confounding, participant selection, classification of intervention, deviations from intended intervention, missing data, outcome measurement, and selective reporting. We appraised each study using a dual reviewer system, in which two blinded reviewers (RU, HA, MA) appraised each included study and provided a rationale for each domain of the ROBINS-I tool. A third blinded reviewer (MA, RU, or HA) then read and evaluated the rationales to create a final appraisal for each study, which was visualized with the *robvis* web-based R programming language package (McGuinness and Higgins, 2021). Additionally, funding sources, reported conflicts of interest, and author-reported limitations extracted for each study were further used to support interpretation of the evidence base and to support interpretation of between-study variation.

## 3 Results

### 3.1 Literature search results

**FIGURE 1:**
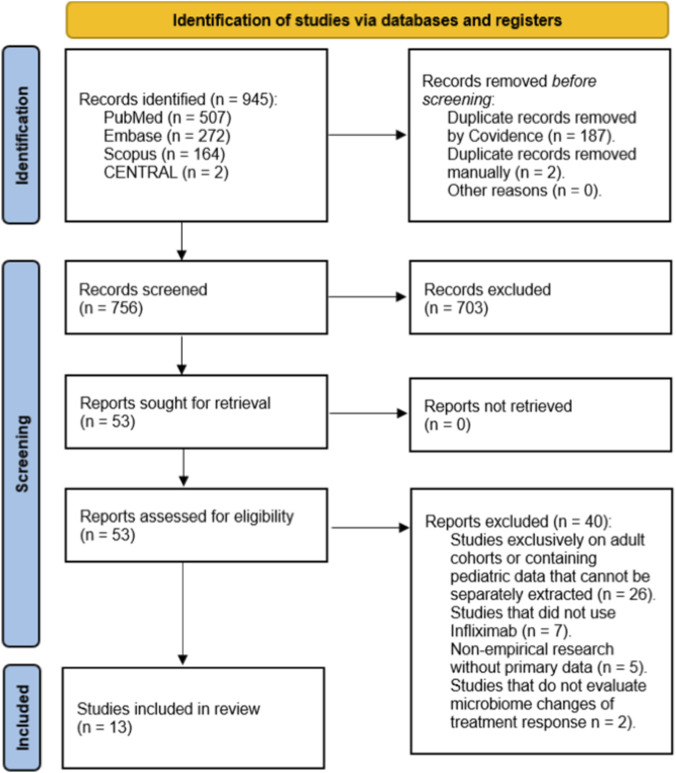
A PRISMA flow chart detailing the inclusion/exclusion process for the 945 identified records, culminating in 13 studies being included in the current review (Page et al., 2021).

The study selection process is visualized in Figure 1 via a PRISMA flow chart (Page et al., 2021). The search of the four databases (PubMed, Scopus, Embase, and Cochrane CENTRAL) yielded 945 study results in total (PubMed: 507 results; Embase: 272 results; Scopus: 164 results; CENTRAL: two results). The Covidence software automatically removed 187 duplicates, while reviewers (MA, HA, RU) manually identified and removed two additional study duplicates prior to the screening stage. Screening of 756 studies was initiated with title-and-abstract screening according to inclusion/exclusion criteria displayed in Box 1, with 703 studies determined to be irrelevant at this stage. All 53 studies that entered the full-text review were successfully retrieved for the full-text, with 40 studies excluded at this stage (26 studies were excluded due to no extractable pediatric patient data; seven studies were excluded due to non-use of IFX; five studies were excluded due to being non-empirical or/and containing no primary data; two studies were excluded due to having outcomes not relevant to the review objectives). Finally, 13 studies (Carlsen et al., 2024; Kowalska-Duplaga et al., 2020; Höyhtyä et al., 2023; Wang et al., 2021; Wang et al., 2017; Taylor et al., 2020; Stein et al., 2022; Salamon et al., 2020; Lv et al., 2024; Kowalska-Duplaga et al., 2019; Kolho et al., 2015; Hurych et al., 2024; Ventin-Holmberg et al., 2022) were deemed to match the inclusion criteria of this review and incorporated in the body of evidence for this systematic review.

### 3.2 Study characteristics

The characteristics of the 13 included studies (Carlsen et al., 2024; Kowalska-Duplaga et al., 2020; Höyhtyä et al., 2023; Wang et al., 2021; Wang et al., 2017; Taylor et al., 2020; Stein et al., 2022; Salamon et al., 2020; Lv et al., 2024; Kowalska-Duplaga et al., 2019; Kolho et al., 2015; Hurych et al., 2024; Ventin-Holmberg et al., 2022), which were published between 2015 and 2024, are summarized in Table 1. One study was conducted in Denmark (Carlsen et al., 2024), three in Finland (Höyhtyä et al., 2023; Kolho et al., 2015; Ventin-Holmberg et al., 2022), three in Poland (Kowalska-Duplaga et al., 2020; Salamon et al., 2020; Kowalska-Duplaga et al., 2019), three in China (Wang et al., 2021; Wang et al., 2017; Lv et al., 2024), one in the Czech Republic (Hurych et al., 2024), one in the United Kingdom (Taylor et al., 2020), and one was an Israel/United States multicentre collaboration (Stein et al., 2022), predominantly in pediatric hospitals, clinics, and tertiary-care gastroenterology settings. Across the included studies, study-level IFX-exposed cohorts comprised 242 children with IBD (136 males and 98 females; two studies did not report IFX-specific sex breakdowns), with the populations heavily weighted toward CD (at least 211 patients had CD), 17 UC and nine IBD-U patients alongside an additional five cases described as UC or IBD-U. Reported ages ranged approximately from 2 to 21 years (with the ages above 18 being due to longitudinal follow-up of pediatric patients into young adulthood), with most studies centered on early adolescence. When reported, IFX was most commonly administered at 5 mg/kg, typically using a standard induction regimen at weeks 0, 2, and 6, followed by maintenance infusions every 4–12 weeks, where applicable. Regarding biospecimens, the literature was overwhelmingly based on stool/fecal sampling, with at least 1462 analyzed biospecimen samples reported across the studies that provided sample counts; only two studies (Wang et al., 2021; Kolho et al., 2015) included blood and stool samples (though blood samples were not analyzed for microbiology), with no included study utilizing mucosal biopsy sampling.

**TABLE 1:** Study characteristics of the 13 included studies (Carlsen et al., 2024; Kowalska-Duplaga et al., 2020; Höyhtyä et al., 2023; Wang et al., 2021; Wang et al., 2017; Taylor et al., 2020; Stein et al., 2022; Salamon et al., 2020; Lv et al., 2024; Kowalska-Duplaga et al., 2019; Kolho et al., 2015; Hurych et al., 2024; Ventin-Holmberg et al., 2022) evaluating infliximab-associated microbiome outcomes in pediatric IBD.

Study (author, year)	Country/Setting	Design	Biologic used/Dose	IFX-focused cohort	Age, y (mean/median; range)	Sex	IBD phenotype (CD/UC/ibd-u)	Comparator/Treatment phase	Biospecimen/Platform
Carlsen et al. (2024)	Denmark; pediatric IBD clinic	Longitudinal observational cohort	IFX; 5 mg/kg	25 IFX-treated children	Median 14.4 (IQR 12.5–16.0); range 10–17	14 M/11 F	CD 16; UC 9; IBD-U 0	Maintenance-cycle follow-up across weeks since last infusion	Stool; 16 S rRNA (V1-V2)
Höyhtyä et al. (2023)	Finland; pediatric hospital	Prospective observational cohort	IFX; 4.5–5.5 mg/kg (1 patient 7.6 mg/kg)	29 IFX-treated children	Median 14; range 6.3–20	16 M/13 F	CD 17; UC 6; IBD-U 6	Induction; baseline, week 2, and week 6 remission vs no remission	Stool; 16 S rRNA (V3-V4) + qPCR
Wang et al. (2017)	China; pediatric hospital	Prospective longitudinal observational cohort	IFX; 5 mg/kg IV	11 IFX-treated children (+ 16 healthy controls)	Mean 11; range 4–17	4 M/7 F (IFX subgroup)	CD 11; UC 0; IBD-U 0	Pre-IFX vs on-therapy; sustained vs non-sustained response	Stool; 16 S rRNA (V3-V4) + PICRUSt/KEGG
Ventin-Holmberg et al. (2022)	Finland; pediatric hospital	Prospective longitudinal observational cohort	IFX; dose NR	30 IFX-treated children	Median 14–15 by response group; range 6–18	21 M/9 F	CD 25; UC 2; IBD-U 3	Induction; baseline, week 2, and week 6 responders vs non-responders	Stool; 16 S rRNA (V3-V4) + fungal ITS1
Kowalska-Duplaga et al. (2020)	Poland; pediatric clinic	Prospective cohort study	IFX (Remsima®); 5 mg/kg	18 IFX-treated children (+ 18 healthy controls)	Mean 13.36 ± 3.71; range 2–18	11 M/7 F	CD 18; UC 0; IBD-U 0	Induction; pre- vs post-IFX plus healthy controls	Stool; 16 S rRNA (V3-V4)
Hurych et al. (2024)	Czech Republic; multicentre pediatric hospitals	Prospective cohort study	Anti-TNFα cohort (18 IFX +1 adalimumab); IFX dose 5 mg/kg	18 IFX-treated children extracted within anti-TNFα cohort	Median 12.9 (IQR 9.1–15.0); range 9.2–15	15 M/22 F (anti-TNFα cohort reported overall)	CD 18 in IFX subgroup; UC 0; IBD-U 0	Induction + maintenance anti-TNFα cohort; IFX subgroup extracted where possible	Stool; 16 S (V4) + metabolomics
Salamon et al. (2020)	Poland; pediatric hospital	Prospective cohort study	IFX (Remsima®); 5 mg/kg	13 IFX-treated children	Mean 13.09 ± 3.76; range NR	7 M/6 F	CD 13; UC 0; IBD-U 0	Induction; baseline vs post-3rd dose	Stool; targeted qPCR
Lv et al. (2024)	China; children’s hospital	Prospective cohort study	IFX; dose NR	4 IFX-treated children within total pediatric CD cohort of 11	Median 13; range 11–15	8 M/3 F (total CD cohort)	CD 4 in IFX subgroup UC 0; IBD-U 0	Induction; pre-treatment vs remission post-IFX (IFX subgroup)	Stool; shotgun metagenomics + miRNA profiling
Stein et al. (2022)	Israel and United States of America; multicentre pediatric gastroenterology hospitals	Prospective cohort study	IFX; dose NR	11 anti-TNFα withdrawal patients analyzed for remission/relapse	Mean 16; range 13–21	12 M/6 F (full cohort)	CD 11; UC 0; IBD-U 0	Withdrawal; baseline to 52 weeks drug-free remission vs relapse	Stool; shotgun metagenomics
Taylor et al. (2020)	United Kingdom; multicentre pediatric hospitals	Prospective cohort study	IFX; dose NR	20 IFX-treated analysed participants (10 low FC; 10 high FC)	Age NR	NR	CD 20; UC 0; IBD-U 0	Maintenance cross-sectional; mucosal healing (low FC) vs inflammation (high FC)	Stool; 16 S rRNA (V1-V2) + metabolomics/proteomics
Kowalska-Duplaga et al. (2019)	Poland; single-centre pediatric gastroenterology clinic	Prospective cohort study	IFX (Remsima®); 5 mg/kg	13 IFX-treated children	Mean 11.4; median 10; range NR	7 M/6 F	CD 13; UC 0; IBD-U 0	Induction; baseline vs 4 weeks after 3rd dose	Stool; *Candida* qPCR
Wang et al. (2021)	China; multicentre pediatric hospitals	Prospective cohort study	IFX; 5 mg/kg (escalated to 10 mg/kg if needed)	18 IFX-treated children within total CD cohort of 29	Median 13 (IQR 11–14)	21 M/8 F (total CD cohort)	CD 18 in IFX subgroup; UC 0; IBD-U 0	Pre/post IFX; sustained vs non-sustained response	Stool ± blood markers; 16 S + ITS2 + metabolomics
Kolho et al. (2015)	Finland; tertiary care hospital	Retrospective cohort study	Anti-TNFα cohort (31 IFX +1 adalimumab); dose NR	32 anti-TNFα-treated children	Age NR for IFX subgroup	NR	CD 27; UC/IBD-U combined 5 (separate counts NR)	Induction + maintenance anti-TNFα; responders vs non-responders	Stool + blood; HITChip phylogenetic microarray + qPCR

Abbreviations: CD, Crohn’s disease; UC, ulcerative colitis; IBD-U, inflammatory bowel disease-unclassified; IFX, infliximab; NR, not reported; TNFα, tumor necrosis factor α; FC, fecal calprotectin; qPCR, quantitative polymerase chain reaction; IV, intravenous; M, male; F, female.

### 3.3 Evidence outcomes

Table 2 provides a structured overview of the evidence outcomes reported across the 13 included studies (Carlsen et al., 2024; Kowalska-Duplaga et al., 2020; Höyhtyä et al., 2023; Wang et al., 2021; Wang et al., 2017; Taylor et al., 2020; Stein et al., 2022; Salamon et al., 2020; Lv et al., 2024; Kowalska-Duplaga et al., 2019; Kolho et al., 2015; Hurych et al., 2024; Ventin-Holmberg et al., 2022) by organizing findings into the domains of alpha diversity, beta diversity, taxonomic findings, functional/metabolic outcomes, and clinical/biochemical outcomes. These domains were chosen to capture both microbiome structure and function, alongside treatment-response outcomes relevant to pediatric IFX therapy. Detailed interpretation of the consistencies, divergences, and clinical implications of these results are explored in the Discussion.

**TABLE 2:** Study-specific quantitative synthesis of alpha diversity, beta diversity, taxonomic, functional/metabolic, and clinical/biochemical outcomes across the captured evidence (n = 13 studies) (Carlsen et al., 2024; Kowalska-Duplaga et al., 2020; Höyhtyä et al., 2023; Wang et al., 2021; Wang et al., 2017; Taylor et al., 2020; Stein et al., 2022; Salamon et al., 2020; Lv et al., 2024; Kowalska-Duplaga et al., 2019; Kolho et al., 2015; Hurych et al., 2024; Ventin-Holmberg et al., 2022).

Outcome domain	Outcome subdomain	Synthesized observed effect	Representative studies	Study-specific quantitative findings
Alpha diversity	Shannon/overall alpha diversity	Induction studies generally showed improved or normalized alpha diversity, whereas maintenance-cycle analyses suggested declining diversity with longer time since infusion; several response-stratified studies found no between-group difference	Carlsen et al. (2024)	Shannon diversity decreased as time since last IFX infusion increased (β = −0.018, *p =* 0.036)
			Wang et al. (2017)	Observed OTUs, Shannon, Simpson, and inverse Simpson were lower pre-IFX than in healthy controls; all improved post-IFX, but exact effect sizes were not reported
			Kowalska-Duplaga et al. (2020)	Shannon was lower pre-IFX vs controls (*p =* 0.003; adj *p =* 0.010); paired pre to post Shannon increased (median difference 0.29, *p =* 0.043)
			Lv et al. (2024)	Authors reported increased alpha diversity after IFX in the remission group (n = 4), but no index-specific effect size was reported
	Richness/observed OTUs/ASVs	Richness improved in some induction cohorts but was not consistently associated with response status or weeks since infusion	Kowalska-Duplaga et al. (2020)	Observed OTUs were lower pre-IFX vs controls (*p =* 0.007; adj *p =* 0.015), increased pre to post (*p =* 0.010; adj *p =* 0.015), and increased in paired analysis (median difference 18.5, *p =* 0.007)
			Carlsen et al. (2024)	Observed ASVs showed no significant association with weeks since last infusion
			Höyhtyä et al. (2023)	Richness ranged from 30 to 126 OTUs (median 72); no remission vs no-remission difference at baseline, week 2, or week 6
			Ventin-Holmberg et al. (2022)	Neither bacterial nor fungal richness differed significantly between responders and non-responders at baseline, week 2, or week 6
			Taylor et al. (2020)	Species richness did not differ between low- and high-calprotectin groups
	Simpson/inverse Simpson/other alpha indices	Alternative alpha-diversity metrics showed variable results across cohorts, with some induction-response signals but generally limited reproducible between-group differences	Kolho et al. (2015)	Inverse Simpson diversity increased in responders (n = 6) but not non-responders (n = 5); at week 6, diversity was higher in responders than non-responders (*p <* 0.01)
			Hurych et al. (2024)	Anti-TNFα exposure was weakly associated with a higher Simpson index in CD (*p =* 0.024), while all alpha indices increased over time overall (p values 0.003 to <10^–6^)
			Höyhtyä et al. (2023)	Inverse Simpson ranged from 1.1 to 22 (median 6.6), with no remission-group differences across induction
			Wang et al. (2021)	Shannon, Chao1, Simpson, and inverse Simpson did not differ significantly between CD and healthy controls or between pre- and post-IFX samples
			Taylor et al. (2020)	Pielou’s evenness and Shannon index both showed no significant difference between mucosal healing and inflammation groups
Beta diversity	Community composition pre- vs post-IFX	Several induction studies showed significant restructuring of community composition after IFX, but the magnitude and persistence of these shifts varied by analytic method and biological compartment	Wang et al. (2017)	Bray-Curtis, weighted UniFrac, and unweighted UniFrac showed pre-IFX samples separated from controls; post-IFX samples shifted toward controls (PERMANOVA *p =* 0.001)
			Kowalska-Duplaga et al. (2020)	Controls differed from CD pre-IFX across all beta metrics (all adj p ≤ 0.003); pre- vs post-IFX differences were significant for Jaccard (adj *p =* 0.030), Bray-Curtis (adj *p =* 0.033), and unweighted UniFrac (adj *p =* 0.014), but not weighted UniFrac (adj *p =* 0.121)
			Wang et al. (2021)	Bacterial beta diversity differed between CD and healthy controls (Bray-Curtis PERMANOVA *p =* 0.0003, *R* ^2^ = 0.202); fungal beta diversity also differed (*p =* 0.036, *R* ^2^ = 0.0937). Pre- vs post-IFX bacterial composition did not shift significantly (*p =* 0.42, *R* ^2^ = 0.0315), whereas fungal composition did (*p =* 0.048, *R* ^2^ = 0.0937)
			Lv et al. (2024)	Bray-Curtis beta diversity differed between active disease and remission states (PERMANOVA *p <* 0.05), but IFX-specific effect sizes were not reported
	Response/similarity-to-control patterns	Response-related differences were more consistently reflected in composition or similarity-to-control measures than in alpha diversity alone	Kolho et al. (2015)	Similarity to healthy control microbiota increased in responders but not non-responders during induction; by week 6, both diversity and similarity-to-control were higher in responders (*p <* 0.01). Similarity-to-control correlated inversely with calprotectin 3 months later (r = −0.72, *p =* 0.0001)
			Ventin-Holmberg et al. (2022)	Baseline bacterial composition differed between responders and non-responders: responders had higher Clostridia and Bacilli (∼1.2-fold each; FDR <0.001), whereas non-responders had higher Gammaproteobacteria (4.4-fold; FDR <0.001). Baseline fungal composition did not differ significantly
			Taylor et al. (2020)	Weighted UniFrac beta diversity did not differ between low- and high-calprotectin groups (PERMANOVA *p =* 0.33)
	Infusion timing/withdrawal/longitudinal stability	In longitudinal maintenance and withdrawal settings, community composition appeared only weakly related to infusion timing and was often dominated by subject-specific stability	Carlsen et al. (2024)	Aitchison genus composition was weakly associated with weeks since infusion (PERMANOVA *R* ^2^ = 0.004, *p =* 0.047); fecal calprotectin was also associated with overall composition (*R* ^2^ = 0.018, *p =* 0.001)
			Stein et al. (2022)	Baseline CD samples differed from healthy controls (Bray-Curtis PERMANOVA *p =* 0.011), but baseline relapse vs remission groups did not (*p =* 0.29). Within-subject distances were highly correlated over time (*R* ^2^ = 0.74, *p =* 0.001), with no association with time point, diet, or remission status (all p ≥ 0.39)
Taxonomic findings	Bacterial compositional shifts after IFX	Induction-phase studies generally described reductions in dysbiosis-associated Proteobacteria/opportunists and enrichment of taxa linked to a healthier, SCFA-associated ecosystem; maintenance analyses suggested some drift across the infusion cycle	Wang et al. (2017)	Pre-IFX CD showed higher *Enterococcus*, *Klebsiella*, *Streptococcus*, and Veillonella and lower Bacteroidetes/SCFA-associated taxa; after IFX, Enterobacteriaceae, Enterococcaceae, Planococcaceae, and Streptococcaceae declined toward control levels
			Kowalska-Duplaga et al. (2020)	After IFX, Actinomycetales decreased significantly; Enterococcaceae and *Clostridium* remained higher than controls, and the overall dysbiosis index did not normalize (*p =* 0.389)
			Wang et al. (2021)	After IFX, Blautia, *Clostridium* IV, Collinsella, Eubacterium, and Ruminococcus increased, whereas Abiotrophia and Lactococcus decreased
			Carlsen et al. (2024)	As time since infusion increased, Parasutterella rose across the cohort (p.adj = 1 × 10^−10^); in UC, Anaerostipes and Fusicatenibacter also declined and Parasutterella increased (all p.adj = 0.037)
			Salamon et al. (2020)	*Lactobacillus* fermentum was significantly higher at baseline in the IFX group and decreased by the end of induction; other targeted species showed no significant longitudinal change
			Lv et al. (2024)	Remission-state samples showed less Proteobacteria dominance and fewer pathogenic Enterobacteriaceae, with higher Roseburia intestinalis and Dorea spp.; exact IFX-specific effect sizes were not reported
	Fungal findings/mycobiome	The clearest fungal signal was divergence by treatment response, with *Candida* expansion in non-response and more favorable Saccharomyces-related patterns in responders	Ventin-Holmberg et al. (2022)	At week 2, Saccharomycetales was higher in responders (1.03-fold; FDR <0.001). At week 6, *Candida*/C. albicans was 22-fold higher in non-responders (FDR <0.001), while *Saccharomyces*/*S. cerevisiae* was 1.9-fold higher in responders (FDR <0.001)
			Kowalska-Duplaga et al. (2019)	*Candida* abundance decreased significantly after IFX induction (*p =* 0.045); post-treatment *Candida* levels were no longer significantly different from controls (*p =* 0.39)
			Wang et al. (2021)	Galactomyces increased after IFX; compared with healthy controls, CD samples had higher Alternaria and Thielavia and lower several other fungal genera
​	Response-associated taxa/predictive signatures	Baseline enrichment of beneficial or butyrate-associated taxa was repeatedly linked to remission or sustained response, whereas inflammatory or dysbiosis-associated taxa were linked to non-response	Ventin-Holmberg et al. (2022)	Responders had higher baseline Faecalibacterium (1.4-fold; FDR <0.001) and Subdoligranulum (1.7-fold; FDR <0.001); non-responders had higher Dialister (1.2-fold; FDR <0.001) and Anaerostipes (2.3-fold; FDR <0.001). By week 6, Bifidobacterium was higher in responders (2.0-fold; FDR = 0.048)
			Höyhtyä et al. (2023)	Baseline remission was associated with higher Bifidobacteriales (∼1.7×; *p =* 0.039), Family XI Incertae Sedis (fold-change 0.10; *p =* 0.028), and Anaerosporobacter (fold-change 0.066; *p =* 0.0013), whereas no remission was associated with Actinomycetales (fold-change 3.39; *p =* 0.018), *Actinomyces* (fold-change 1315; *p =* 0.0077), Parasutterella (fold-change 145; *p <* 0.001), and Parabacteroides (fold-change 8.19; *p =* 0.044)
			Kolho et al. (2015)	Responders had higher baseline Bifidobacterium, *Clostridium* colinum, Eubacterium rectale, uncultured Clostridiales, and *Vibrio*, and lower *Streptococcus* mitis. Biomarker performance was strongest for Bifidobacterium and C. colinum (sensitivity, specificity, PPV, and NPV all 1.0)
			Wang et al. (2017)	Sustained responders showed greater expansion of Blautia, Faecalibacterium, Lachnospira, and Roseburia than non-sustained responders; exact effect sizes were not reported
			Wang et al. (2021)	Baseline sustained-response taxa included Methylobacterium, Sphingomonas, *Staphylococcus*, and *Streptococcus*; non-sustained response was linked to *Clostridium* XI/XVIII, Eggerthella, Lachnospiraceae incertae sedis, Parabacteroides, and *Peptococcus*. After therapy, *Actinomyces* and Atopobium were higher in sustained responders, whereas Dorea and Holdemania were higher in non-sustained responders
			Stein et al. (2022)	No significant individual taxa or gene orthologs differed between baseline and week 52 anti-TNFα remission samples (*p =* 0.74)
Functional/metabolic	Predicted pathway shifts after IFX	Functional inference studies suggested that IFX can move pathway profiles away from inflammatory or dysbiotic signatures and toward more homeostatic metabolic configurations	Wang et al. (2017)	Pre-IFX samples were enriched for xenobiotics biodegradation/metabolism and, at KEGG level 3, transcription factors, secretion system, phosphotransferase system, and pathogenic *Escherichia coli* infection. Post-IFX samples showed no significant KEGG differences vs controls at LEfSe LDA >3.0
			Kowalska-Duplaga et al. (2020)	PICRUSt/KEGG suggested enrichment of α-linolenic acid metabolism, stilbenoid biosynthesis, phosphotransferase system, and ion channels before treatment; after IFX, pathways related to bile acids, protein digestion, and adipocytokine signaling were induced
			Wang et al. (2021)	IFX restored bile-acid metabolism: conjugated bile acids decreased and unconjugated/conjugated and secondary/primary bile-acid ratios increased, alongside enrichment of BSH-producing Blautia/Collinsella and 7α-dehydroxylating *Clostridium* IV/Eubacterium
	SCFAs, bile acids, and other metabolites	The metabolomic literature suggested that inflammatory disease states are marked by depletion of beneficial microbial metabolites, with partial recovery after effective therapy, although not all cohorts demonstrated anti-TNFα-specific SCFA changes	Wang et al. (2021)	Baseline CD showed lower acetic, butyric, and propanoic acids plus lower deoxycholic, hyodeoxycholic, and lithocholic acids; after IFX, several amino acids and inflammatory fatty acids decreased, while azelaic acid increased
			Taylor et al. (2020)	Pentanoate (valerate) was 1.35-fold higher in the low-calprotectin group, whereas lysine was 1.54-fold higher in the high-calprotectin group. Proteomics and bile-acid comparisons were not significant
			Hurych et al. (2024)	Anti-TNFα exposure was not associated with total SCFA concentration (*p =* 0.44), SCFA/branched-chain FA ratio (*p =* 0.19), or any individual SCFA after multiple-testing correction (all corrected *p =* 1.0)
	Functional predictors of response/relapse	Baseline functional and metabolomic signatures appeared more promising than broad diversity metrics for predicting durable response, non-response, or relapse	Stein et al. (2022)	At week 52, relapse was associated with higher ABC transporter (*p =* 0.04) and quorum-sensing pathways (*p =* 0.001) and lower amino-sugar/nucleotide-sugar metabolism, galactose metabolism, and glycan degradation pathways (all *p =* 0.03). A baseline pathway model predicted relapse with ∼80% accuracy
			Wang et al. (2021)	Sustained response was associated with higher baseline glycine, linoleic acid, and L-lactic acid; non-sustained response had higher N-acetylserotonin, methylglutaric acid, adipic acid, 4-aminohippuric acid, citramalic acid, isovaleric acid, and nicotinic acid
			Ventin-Holmberg et al. (2022)	Responders were enriched at baseline for butyrate-associated taxa (Clostridia, Faecalibacterium, Subdoligranulum), supporting an anti-inflammatory SCFA-producing functional profile, although no direct metabolite quantification was reported
Clinical/biochemical	Clinical activity/remission	Across induction studies, IFX was consistently associated with marked clinical improvement, although maintenance or comparator-based cohorts sometimes showed weaker relationships between symptoms and microbiome markers	Kowalska-Duplaga et al. (2020)	PCDAI fell from 44.75 ± 19.44 to 4.86 ± 4.49 (*p <* 0.05); 16/18 patients (88%) achieved remission
			Kowalska-Duplaga et al. (2019)	PCDAI fell from 47.5 ± 16.43 (range 5–60) to 9.04 ± 6.5 (range 0–20) after induction (*p =* 0.00)
			Salamon et al. (2020)	PCDAI decreased from 47.5 ± 16.43 to 9.04 ± 6.50 after IFX (*p <* 0.001)
			Lv et al. (2024)	All IFX-treated patients (4/4; 100%) entered remission; PCDAI range improved from 12.5 to 42.5 to 0–5
			Wang et al. (2017)	All IFX-treated children initially entered remission, but only 4/11 maintained sustained remission through follow-up
			Ventin-Holmberg et al. (2022)	Biochemical induction response rate was 12/30 (40%) achieving fecal calprotectin <100 μg/g by week 6; 18/30 (60%) were non-responders
			Stein et al. (2022)	After anti-TNFα withdrawal, 7/11 (63.6%) remained in drug-free remission at 52 weeks
			Carlsen et al. (2024)	PCDAI did not differ between low- and high-calprotectin groups (*p =* 0.5)
			Carlsen et al. (2024)	No formal remission-rate analysis was reported, but UC symptom score was negatively associated with Shannon diversity (*p =* 0.012) and observed ASVs (*p =* 0.0007)
​	Biochemical inflammation markers	Fecal calprotectin and systemic inflammatory markers generally improved with successful IFX treatment and often tracked more closely with microbiome signatures than broad diversity measures did	Kowalska-Duplaga et al. (2020)	Fecal calprotectin fell from 1920.44 ± 1326.73 to 629.17 μg/g (*p <* 0.05)
			Ventin-Holmberg et al. (2022)	Median fecal calprotectin (µg/g) was lower in responders than non-responders at baseline (360 (7–1317) vs 769 (55–6293)), week 2 (25 (<5–496) vs 456 (36–1876)), and week 6 (41 (5–89) vs 399 (162–2142))
			Höyhtyä et al. (2023)	Baseline fecal calprotectin was lower in the remission group (mean 326 μg/g (38–1317)) than in the no-remission group (mean 1038 μg/g (186–6293))
​	​	​	Kolho et al. (2015)	Responders showed an approximately 10-fold fall or normalization in fecal calprotectin during induction and maintained levels <200 μg/g at follow-up; non-responders had persistently high levels >200 μg/g
			Hurych et al. (2024)	Fecal calprotectin decreased by a mean of 67%–70% during anti-TNFα treatment; patients requiring anti-TNFα had a median calprotectin of 1424 μg/g vs 219 μg/g in those managed without it
			Taylor et al. (2020)	Calprotectin correlated negatively with Dialister abundance (*p =* 0.0001) and pentanoate (*p =* 0.006), and positively with lysine (*p =* 0.009)
			Wang et al. (2021)	Sustained responders showed significant reductions in CRP, WBC, ESR, and platelets. CRP correlated positively with *Mycoplasma*, Parasutterella, Finegoldia, and *Haemophilus*, and negatively with Blautia, Eggerthella, and Eubacterium
			Salamon et al. (2020)	CRP fell numerically from 11.53 ± 11.41 to 8.13 ± 7.27 mg/dL; correlation between CRP and bacterial abundance was not significant
			Carlsen et al. (2024)	Fecal calprotectin was associated with community composition (*R* ^2^ = 0.018, *p =* 0.001) but not with Shannon diversity or observed ASVs
	Endoscopic/histologic outcomes	Where reported, endoscopic healing improved after induction, but endoscopic or histologic findings were less often linked to distinct microbiome signatures	Kowalska-Duplaga et al. (2020)	SES-CD decreased significantly after IFX induction (*p <* 0.01), and endoscopic response correlated with fecal calprotectin
			Lv et al. (2024)	CDEIS improved alongside PCDAI and calprotectin in the remission group (*p <* 0.05)
			Stein et al. (2022)	Baseline histologic healing did not predict relapse after withdrawal (*p =* 0.62), and endoscopic outcomes were not linked to microbiome differences

Abbreviations: IFX, infliximab; NR, not reported; FA, fatty acid; SCFA, short-chain fatty acid; FC, fecal calprotectin; PCDAI, Pediatric Crohn’s Disease Activity Index; SES-CD, Simple Endoscopic Score for Crohn’s Disease; CDEIS, Crohn’s Disease Endoscopic Index of Severity; SCFA, short-chain fatty acid; PERMANOVA, permutational multivariate analysis of variance; OTU, operational taxonomic units; ASV, amplicon sequence variants.

### 3.4 Quality appraisal

**FIGURE 2:**
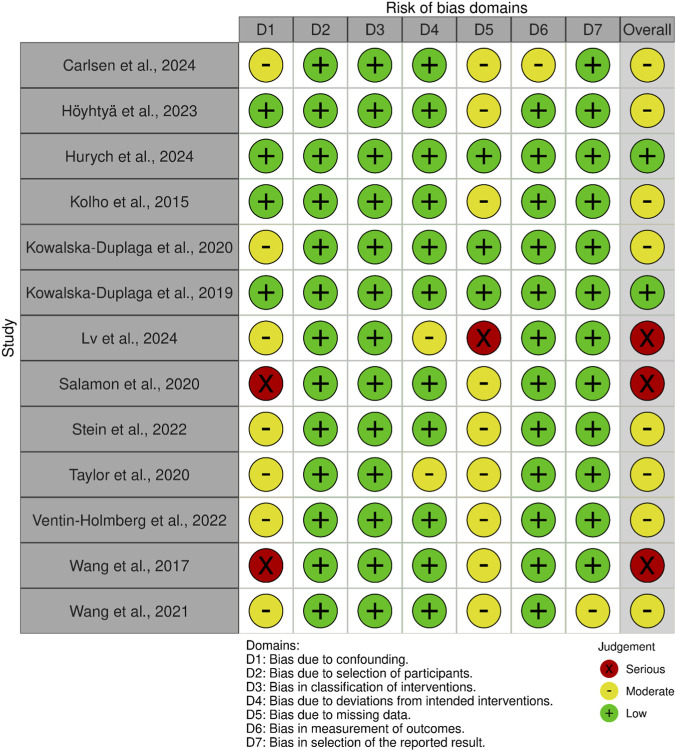
ROBINS-I traffic-light plot of all included studies (n = 13) (Carlsen et al., 2024; Kowalska-Duplaga et al., 2020; Höyhtyä et al., 2023; Wang et al., 2021; Wang et al., 2017; Taylor et al., 2020; Stein et al., 2022; Salamon et al., 2020; Lv et al., 2024; Kowalska-Duplaga et al., 2019; Kolho et al., 2015; Hurych et al., 2024; Ventin-Holmberg et al., 2022).

**FIGURE 3:**
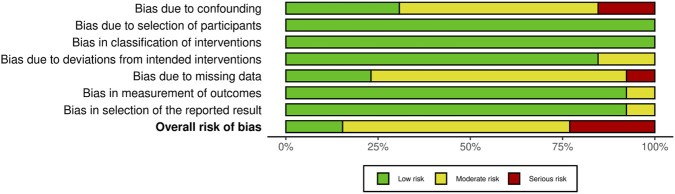
ROBINS-I grouped domain risk-of-bias analysis of all included studies (n = 13) (Carlsen et al., 2024; Kowalska-Duplaga et al., 2020; Höyhtyä et al., 2023; Wang et al., 2021; Wang et al., 2017; Taylor et al., 2020; Stein et al., 2022; Salamon et al., 2020; Lv et al., 2024; Kowalska-Duplaga et al., 2019; Kolho et al., 2015; Hurych et al., 2024; Ventin-Holmberg et al., 2022).

Using the ROBINS-I tool to appraise the 13 included non-randomized studies (Carlsen et al., 2024; Kowalska-Duplaga et al., 2020; Höyhtyä et al., 2023; Wang et al., 2021; Wang et al., 2017; Taylor et al., 2020; Stein et al., 2022; Salamon et al., 2020; Lv et al., 2024; Kowalska-Duplaga et al., 2019; Kolho et al., 2015; Hurych et al., 2024; Ventin-Holmberg et al., 2022), the overall risk of bias was judged as low in 2/13 (15.4%) (Kowalska-Duplaga et al., 2019; Hurych et al., 2024), moderate in 8/13 (61.5%) (Carlsen et al., 2024; Kowalska-Duplaga et al., 2020; Höyhtyä et al., 2023; Wang et al., 2021; Taylor et al., 2020; Stein et al., 2022; Kolho et al., 2015; Ventin-Holmberg et al., 2022), and serious in 3/13 (23.1%) (Wang et al., 2017; Salamon et al., 2020; Lv et al., 2024), with no studies rated as containing a critical risk of bias as depicted in Figures 2, 3. Across domains, selection of participants (Domain 2 (D2)) and classification of interventions (D3) were consistently low risk, and most studies were also low risk for deviations from intended interventions (D4), outcome measurement (D6), and selective reporting (D7). The main drivers of higher overall ratings were bias due to confounding (D1) (frequently only partially addressed, with common confounding factors such as unmeasured diet/lifestyle factors, baseline disease severity differences, and concomitant medications like antibiotics) and bias due to missing data (D5) (often reflecting attrition/exclusions and incomplete longitudinal sampling, raising the possibility of survivorship/relapse-related bias). These same issues contribute to variation between studies as studies differed in how well they controlled key confounders (especially diet, antibiotic exposure, and baseline activity) in population/clinical contexts, and in study conduct and data completeness (dropout, exclusions for missingness, declining samples at later timepoints). In addition, microbiome outcomes are particularly sensitive to methodological variation (e.g., sampled biospecimen type, different assays such as 16 S sequencing vs targeted qPCR, and different follow-up timepoints across induction/maintenance), which may influence the magnitude of observed effects even when all studies evaluate IFX-related microbiome change. Findings from our quality appraisal should be used to improve future IFX-based intervention study designs, particularly with a focus on addressing potential confounding variables (D1) and missing data (D5; e.g., from participant drop-outs).

## 4 Discussion

### 4.1 Principal findings

IFX is increasingly being used in pediatric IBD refractory to steroids and/or immunomodulators, with high sustained remission rates (Ashton and Beattie, 2024). Its anti-inflammatory effects may alter the gut ecosystem by reducing TNF-driven inflammation and facilitating mucosal recovery, although the extent of microbiome restoration appears variable (Papamichael et al., 2019). Multiple factors shape IFX’s pharmacokinetics and pharmacodynamics, while emerging evidence suggests that microbiome features may be associated with IFX exposure and response (Hemperly and Vande Casteele, 2018). Across the 13 included studies (Carlsen et al., 2024; Kowalska-Duplaga et al., 2020; Höyhtyä et al., 2023; Wang et al., 2021; Wang et al., 2017; Taylor et al., 2020; Stein et al., 2022; Salamon et al., 2020; Lv et al., 2024; Kowalska-Duplaga et al., 2019; Kolho et al., 2015; Hurych et al., 2024; Ventin-Holmberg et al., 2022), IFX was consistently associated with clinical and biochemical improvement; however, microbiome changes varied by treatment phase, response status, and analytical approach. These findings suggest that IFX is associated with selective microbial restructuring rather than uniform restoration, with reproducible compositional and functional patterns across diverse study designs. Despite methodological variation, consistent signals emerged, including enrichment of short-chain fatty acid–producing taxa in responders and persistence of dysbiosis-associated taxa in non-responders.

### 4.2 Diversity changes related to IFX treatment

Pediatric IBD is characterized by profound gut dysbiosis that impacts the critical maturation of children’s developing microbiome and is associated with a more aggressive disease phenotype, marked by higher inflammatory markers and clinical activity scores (Wang and Zhan, 2025; Ma et al., 2023). Across the captured evidence, IFX was more consistently associated with shifts in community composition and function than with uniform recovery of alpha diversity.

The developmental state of the pediatric microbiome represents a critical biological consideration for interpreting IFX-associated microbiome changes in children with IBD. Unlike adults, the pediatric gut microbiota undergoes dynamic compositional shifts shaped by host development, dietary transitions, environmental exposures, and pubertal maturation, and adult-like ecological stability may not be fully established until late adolescence (Shin et al., 2019; Gaufin et al., 2018; Rodriguez et al., 2015). The gut microbiome also contributes to the developmental education and maturation of mucosal immune networks, including regulatory T cell populations and secretory IgA responses, while these immune pathways reciprocally shape microbial community structure (Qiu et al., 2022; Shan et al., 2022; Gaufin et al., 2018; Rodriguez et al., 2015). In pediatric IBD, baseline dysbiosis is superimposed on this developmentally dynamic ecosystem, with reduced microbial diversity and compositional shifts reported in pediatric cohorts (Fitzgerald et al., 2021; Iliev et al., 2025; Maeder and Nguyen, 2025; Wang and Zhan, 2025; Ma et al., 2023). These overlapping developmental and disease-related factors may amplify inter-individual variability in microbiome composition and reduce the reproducibility of treatment-associated microbiome signals across patients. This may partly explain the inter-study variability in diversity metrics captured by this review and why pediatric patterns should not be directly extrapolated from adult cohorts, underscoring the importance of age-stratified analyses and developmentally informed frameworks in future pediatric microbiome research (Seong et al., 2020; Carlsen et al., 2024; Kowalska-Duplaga et al., 2020; Zhuang et al., 2020; O'Reilly et al., 2023).

#### 4.2.1 Alpha diversity

Alpha diversity reflects gut resilience and the restoration of homeostasis following biological therapy (Vich Vila et al., 2018). An increase in alpha diversity typically mirrors endoscopic mucosal healing (Wang et al., 2021). Across the included studies, IFX demonstrated modest and variable effects on alpha diversity, with patterns differing between induction and maintenance phases, and between responders and non-responders.

During induction, several studies demonstrated partial increases in alpha diversity measures (Kowalska-Duplaga et al., 2020; Höyhtyä et al., 2023; Ventin-Holmberg et al., 2022). Kowalska-Duplaga et al., 2020 (Kowalska-Duplaga et al., 2020) reported a statistically significant increase in Shannon diversity, with a median paired difference of 0.29 (*p =* 0.043) in pediatric CD after induction. OTU based richness was inconsistent, and the inverse Simpson index showed wide inter-study variability, ranging from 1.1 to 22, despite clinical improvement (Carlsen et al., 2024; Kowalska-Duplaga et al., 2020; Höyhtyä et al., 2023; Ventin-Holmberg et al., 2022). These results mirror findings in adult cohorts, with consistently reduced baseline alpha diversity (Abdel-Rahman and Morgan, 2023), whereas post-IFX recovery is variable; as shown through no significant increase in Shannon diversity following IFX induction in one meta analysis on an adult CD cohort (Mah et al., 2023). Together, these observations are more consistent with early clinical benefit occurring alongside immunological improvement rather than uniform restoration of overall microbiome diversity. However, these observational data cannot determine whether microbiome recovery is a driver of response or a downstream marker of reduced inflammation (Wang et al., 2021).

Maintenance-phase data suggested a different trajectory. The longest follow-up study captured (2 Years) by Carlsen et al. (Carlsen et al., 2024), reported a significant cyclical decline in Shannon diversity as the interval between infusions progressed (*p =* 0.036), suggesting that the stabilizing effect of IFX on the microbiome may be transient. This pattern is similar to adult data by Seong et al. (Seong et al., 2020), that found no significant changes in microbial composition, richness, or diversity between week one and week seven in maintenance phase. Additionally, Seong et al. found that trough levels of ≥5 μg/mL were associated with a significant increase in richness, suggesting that IFX pharmacokinetic efficiency, not only the treatment stage, may drive increases in diversity. No pediatric study in this review collected comparable pharmacokinetic data.

When stratified by treatment response, responders generally showed greater diversity than non-responders, but differences were not consistently significant across studies, and no single metric reliably discriminated between the two groups (Höyhtyä et al., 2023; Taylor et al., 2020; Kolho et al., 2015; Ventin-Holmberg et al., 2022). Kowalska-Duplaga et al. (2020) reported a statistically significant increase in Shannon diversity following induction in CD responders, with a median paired difference of 0.29 (*p =* 0.043), representing the most robust alpha diversity signal captured in this review. In contrast, Höyhtyä et al. (Höyhtyä et al., 2023) and Ventin-Holmberg et al. (Ventin-Holmberg et al., 2022) found no significant difference in richness or inverse Simpson diversity between responders and non-responders groups at induction. Taylor et al. (2020) found no difference in richness, evenness, or Shannon index between mucosal healing and active inflammation groups during maintenance. These findings align with adult literature. Mah et al. (2023) found no significant pooled increase in Shannon diversity between responders and non-responders and concluded that diversity metrics alone lack the level of sensitivity necessary to serve as a biomarker for IFX treatment success.

Collectively, modest increases in alpha diversity may occur during induction, but are transient and poorly correlated with treatment response, particularly over longer follow-up. Instead, community composition and functional outputs can offer a more accurate understanding of microbiome dynamics in IFX-treated pediatric IBD.

#### 4.2.2 Beta diversity

Beta diversity quantifies the degree of dissimilarity in microbial composition between samples, reflecting how the entire ecological structure differs across individuals or timepoints (Jovel et al., 2016). In contrast to alpha diversity findings, beta diversity analyses revealed more clinically meaningful changes, capturing treatment-associated restructuring of the gut microbiome and providing greater discriminatory power between responders and non-responders.

Multiple induction studies demonstrated significant composition shifts following IFX initiation, even in the absence of substantial changes in alpha diversity (Kowalska-Duplaga et al., 2020; Wang et al., 2021; Wang et al., 2017; Lv et al., 2024; Kolho et al., 2015). Significant shifts toward control-like profiles following IFX induction: *p <* 0.01 (Kolho et al., 2015), *p =* 0.033 (Kowalska-Duplaga et al., 2020), and *p <* 0.05 (Lv et al., 2024), corresponding with findings in adult cohorts. For instance, in a large adult CD cohort (n = 49), Zhuang et al. (Zhuang et al., 2020) reported that responders’ microbiota shifted toward healthy controls while non-responders retained a dysbiosis profile.

Notably, this directional pattern was concordant whether studies compared patients to healthy controls (Kowalska-Duplaga et al., 2020; Wang et al., 2021; Wang et al., 2017; Lv et al., 2024) or responders to non-responders (Höyhtyä et al., 2023; Taylor et al., 2020; Ventin-Holmberg et al., 2022). The one exception was Taylor et al. (2020), who examined beta diversity during maintenance rather than induction, and found no significant difference in weighted UniFrac between mucosal healing and inflammation groups (PERMANOVA *p =* 0.33), suggesting beta diversity may lose discriminatory power once patients reach maintenance. This contrasts with data from adult cohorts, such as Seong et al. (2020), who reported that mucosal healing and non-mucosal healing groups distributed separately on principal coordinate analysis (PCoA), with significantly higher beta diversity in mucosal healing patients, and differed in abundance of *Faecalibacterium prausnitzii*. This discrepancy between pediatric and adult findings may reflect differences in statistical power, and greater inter-individual compositional variability inherent to the developing pediatric microbiome, that is less mature and more sensitive to perturbation (Rodriguez et al., 2015).

The microbiome-modulating effects of IFX must be interpreted within the broader landscape of IBD therapeutics, as drug classes interact with the gut microbiome through fundamentally distinct mechanisms. Conventional agents such as 5-aminosalicylic acid (5-ASA) and azathioprine, are directly metabolized by gut bacteria: 5-ASA is inactivated by bacterial arylamine N-acetyltransferases, and azathioprine is converted to 6-thioguanine nucleotides with up to tenfold variation between individuals depending on microbiome composition (Lazarevic et al., 2022). These conventional agents may therefore produce taxonomic effects that are less structured than those of biological therapies (O'Reilly et al., 2023).

By contrast, IFX-associated microbial shifts may occur indirectly through reduced mucosal inflammation and partial restoration of ecological conditions that favor commensal taxa. This interpretation remains inferential, because most included studies cannot separate treatment-driven ecological recovery from microbiome features preceding IFX response. Among biologics, anti-TNF therapies share broadly similar associative microbiome patterns; adalimumab responders paralleled pediatric and adult IFX responses, by also demonstrating decreased *Proteobacteria* and increased *Lachnospiraceae*. (Zhuang et al., 2020; O'Reilly et al., 2023). Vedolizumab, which blocks gut-specific lymphocyte trafficking, appears to show a different profile, where pre-treatment microbiome richness does not predict its response as it does for anti-TNF and ustekinumab (Mah et al., 2023). Collectively, this suggests class-wide anti-TNF microbiome-modulating effects are conserved across age groups, partially shared with ustekinumab, and mechanistically distinct from vedolizumab, with greater compositional specificity than conventional agents. However, further evidence is required for these findings to become established.

### 4.3 Taxonomic shifts and response associated signatures

In IBD-associated dysbiosis, distinct clustering patterns of common bacteria are observed compared with healthy individuals (Sharma et al., 2025). In pediatric IBD, there is a decreased abundance of *Coriobacteriaceae* and *Lachnospiraceae* and decreased diversity of *Ruminococcaceae, Bifidobacteriaceae,* and butyrate-producing families (Kolho et al., 2015; Maukonen et al., 2015). There is also an over-abundance of *Escherichia coli* (phylum *Proteobacteria*), driving T cell-mediated mucosal barrier effects (Wang and Zhan, 2025).

Across the captured studies, IFX treatment was associated with shifts away from dysbiosis-linked taxonomic patterns.

After induction, the most consistent shift amongst responders was a decline in the dominance of *Proteobacteria*, with reductions in *Klebsiella, Streptococcus,* and *Veillonella*, and expansion of SCFA-associated genera including *Roseburia intestinalis, Blautia, Faecalibacterium*, and *Lachnospira* (Kowalska-Duplaga et al., 2020; Wang et al., 2017; Lv et al., 2024). Ventin-Holmberg et al. (Ventin-Holmberg et al., 2022) reported higher baseline of *Faecalibacterium* abundance (1.4-fold, FDR <0.001) and *Subdoligranulum* (1.7-fold, FDR <0.001) in responders, with *Bifidobacterium* becoming further enriched by week 6 (2.0-fold, FDR = 0.048). Höyhtyä et al. (Höyhtyä et al., 2023) linked week-6 remission to higher baseline *Bifidobacteriales* (∼1.7-fold, p = 0.039), and Wang et al., 2017 (Wang et al., 2017) reported greater expansion of SCFA-associated genera *(Faecalibacterium, Blautia, Lachnospira, Roseburia*) in sustained responders. Kolho et al. (Kolho et al., 2015) reported that several taxa, including *Bifidobacterium* and *Eubacterium rectale*, achieved perfect predictive performance in their cohort (sensitivity, specificity, PPV, NPV all = 1.0); however, these metrics likely reflect overfitting due to a small sample size and require validation in larger cohorts.

Across the included studies, several taxa which changed during treatment were also reported as enriched at baseline in later responders, suggesting taxonomic and response associated signals may overlap. However, these findings may only be associative as the studies are unable to determine whether these taxa contribute to treatment associated response or a pre-existing inflammatory burden of the microbiome.

Non-responders showed an inverse pattern, with limited expansion of beneficial SCFA-producing bacteria alongside enrichment of *Gammaproteobacteria* (4.4-fold; FDR <0.001) (Ventin-Holmberg et al., 2022) and other pro-inflammatory or dysbiosis-linked organisms, including *Actinomycetales*, *Actinomyces*, *Parasutterella*, *Parabacteroides*, *Dialister*, and *Anaerostipes* (Höyhtyä et al., 2023; Ventin-Holmberg et al., 2022). Fungal findings followed a similar pattern; in responders, *Candida* burden significantly decreased after IFX induction (*p* = 0.045) and became closer to controls (*p* = 0.39), whereas non-responders showed a 22-fold increase of *Candida* by week 6 (FDR<0.001) (Kowalska-Duplaga et al., 2019; Ventin-Holmberg et al., 2022). *Candida* can promote pro-inflammatory cytokine expression and intensify intestinal inflammation, making the observed overabundance clinically relevant (Zhou et al., 2021).

These pediatric findings align with adult cohorts. Sanchis-Artero et al. (Sanchis-Artero et al., 2021) reported greater diversity and higher *Faecalibacterium prausnitzii* in responders, with *F. prausnitzii* to *E. coli* ratio predicting response with an AUC of 0.87, while non-responders showed *Proteobacteria* enrichment. Across pediatric and adult data, microbiomes enriched in SCFA-producing taxa are associated with favorable therapeutic response, whereas *Proteobacteria*-dominated dysbiosis associated with treatment failure. This suggests that favorable IFX outcomes in pediatric IBD are associated with baseline ecological composition, particularly SCFA producing and microbiota-stabilizing taxa, and subsequent microbial reorganization during inflammatory improvement (Seong et al., 2020). Predictive modeling is still underexplored in pediatric populations, and future studies should integrate microbial composition with functional, pharmacokinetic, endoscopic, and clinical biomarkers before microbiome-informed precision medicine can be recommended.

### 4.4 Functional and metabolomic outcomes

Of the thirteen included studies, seven reported functional or metabolomics outcomes (Kowalska-Duplaga et al., 2020; Wang et al., 2021; Wang et al., 2017; Taylor et al., 2020; Stein et al., 2022; Hurych et al., 2024; Ventin-Holmberg et al., 2022), extending insight beyond taxonomy and diversity. Those studies collectively suggest that IFX treatment was associated with functional/metabolic reprogramming in addition to taxonomic restructuring. A recurring trend was the restoration of metabolic pathways associated with SCFA production and bile acid metabolism; pathways central to intestinal epithelial integrity and mucosal immune regulation. Wang et al., 2021 (Wang et al., 2021) reported that IFX induction shifted metabolomic profiles toward healthy controls, including changes in bile-acid metabolism, bile-salt hydrolysis, and secondary bile-acid production, mirroring Kowalska-Duplaga et al., 2020 (Kowalska-Duplaga et al., 2020). Similarly, Stein et al. (Stein et al., 2022) reported enrichment of inflammatory signaling pathways; ABC transporters (P = 0.04) and quorum-sensing pathways (P = 0.001) in children who subsequently relapsed after anti-TNFα withdrawal, suggesting persistent functional dysbiosis despite apparent clinical remission.

Certain studies, such as Hurych et al. (Hurych et al., 2024) reported no significant changes in total SCFA concentrations after IFX, suggesting that metabolomic recovery is not uniform, possibly reflecting the reliance on stool rather than mucosal samples. Functional pathway shifts were observed even when alpha diversity remained unchanged in some cohorts (Wang et al., 2017; Taylor et al., 2020; Stein et al., 2022), suggesting that metabolic recovery may precede detectable changes in richness.

Furthermore, adult literature is concordant, Ding et al. (Ding et al., 2020) reported metabolic models profiles predicting anti-TNFα response in adults with CD, with ROC values up to 0.81 (fecal bile acids) and 0.94 (fecal lipid markers). Similarly, Zhuang et al. (Zhuang et al., 2020) demonstrated that anti-TNFα therapy in adults was associated with predicted Kyoto Encyclopedia of Genes and Genomes (KEGG) pathway changes involving SCFA metabolism and beneficial taxa enrichment. Altogether, data suggests that functional recovery may represent an early signal of therapeutic response, preceding or occurring independently of broader taxonomic restructuring.

### 4.5 Clinical outcomes

Despite heterogeneous microbiome metrics, IFX consistently produced rapid improvements in disease activity scores and inflammatory biomarkers, with the largest benefit observed at induction. Most studies (Kowalska-Duplaga et al., 2020; Salamon et al., 2020; Lv et al., 2024; Kowalska-Duplaga et al., 2019) showed marked early reductions in PCDAI and high remission rates. Wang et al., 2017 (Wang et al., 2017), found that all patients initially entered remission, but only a small subset maintained sustained remission over follow-up.

This divergence becomes clearer in maintenance and longitudinal studies, (Carlsen et al., 2024; Wang et al., 2017; Taylor et al., 2020; Stein et al., 2022), where patients often remained in clinical remission despite static or even declining microbial diversity, suggesting that microbiome normalization may lag after inflammatory control. This is consistent with adult IFX literature, where longer-term outcomes are more dependent on deeper therapeutic targets (such as mucosal healing), and adequate drug exposure, which can be affected by factors like the development of anti-drug antibodies (ADA) (Seong et al., 2020; Papamichael et al., 2018)

When stratified by response, biochemical markers like fecal calprotectin emerge as a more consistent indicator of treatment response compared to microbiome diversity. Multiple studies reported substantial reductions in fecal calprotectin following IFX therapy (Kowalska-Duplaga et al., 2020) (Hurych et al., 2024), and lower baseline or early-treatment calprotectin levels could predict subsequent remission (Höyhtyä et al., 2023; Ventin-Holmberg et al., 2022). Notably, only Ventin-Holmberg et al. (Ventin-Holmberg et al., 2022) combined microbiome features with fecal calprotectin in a predictive model, achieving an AUC of approximately 0.89, highlighting the value of integrated biomarker approaches. Collectively, findings support a model in which IFX rapidly suppresses inflammation while microbiome recovery and long-term disease stability follow more heterogeneous trajectories, reinforcing the role of microbiome profiling as an adjunct, rather than a replacement, to established clinical and biochemical markers.

As for the unresolved question, of whether microbiome recovery itself confers protection against later relapse, Stein et al. (2022), the only longitudinal pediatric anti-TNFα withdrawal cohort in this review, reported that gene function and taxonomic abundance remained stable in both relapse and sustained remission groups, with predictive value residing in baseline functional pathways rather than in post treatment recovery. Adult data showed a similar mix, Sakurai et al. (2020) observed post-anti-TNFα microbiome composition shifted in the patients that later relapsed, but remained stable in remission groups, implying post treatment trajectory may carry prognostic information; however, this study was conducted in a small cohort (n = 9) and not replicated at scale. To date, no study demonstrated that adjuvant microbiome targeted interventions, like fecal microbial transplantation, or dietary modifications, would improve long term outcomes beyond IFX treatment alone. Leaving the status of microbiome recovery as a future direction for research.

### 4.6 Sampling matrix and timing

All studies included depended on stool sampling (Carlsen et al., 2024; Kowalska-Duplaga et al., 2020; Höyhtyä et al., 2023; Wang et al., 2021; Wang et al., 2017; Taylor et al., 2020; Stein et al., 2022; Salamon et al., 2020; Lv et al., 2024; Kowalska-Duplaga et al., 2019; Kolho et al., 2015; Hurych et al., 2024; Ventin-Holmberg et al., 2022), owing to the ease of collection of stool and its appropriateness for longitudinal sampling; however, stool-based analysis reflects luminal microbial dynamics that are influenced by transient factors such as diet. The sole use of stool specimens amongst the captured studies limits interpretation of mucosa-associated communities that are more directly engaged in barrier function and intestinal inflammation. Pediatric studies incorporating mucosal samples and absolute quantification represent a gap in the literature. In a recent adult study, IFX treatment was reported to increase mucosal and fecal bacterial load during successful treatment, indicating that relative abundance alone may mask true ecological recovery (Ventin-Holmberg et al., 2025). Sample timing also matters: substantial diversity differences were reported between the induction and maintenance phases, and within-cycle changes were observed in diversity and taxa as time since infusion increased in maintenance cycles (Carlsen et al., 2024). Moreover, adult data link microbiome richness to trough levels and mucosal healing (Seong et al., 2020), whereas no pharmacokinetic measures were collected in pediatric studies.

Most included studies solely focused on the induction phase (baseline through week 6) or maintenance, with few samples per patient. These short sampling windows may capture early inflammatory shifts (like the reduction in facultative anaerobes) but cannot capture the re-expansion of SCFA-producing bacteria, which requires sustained mucosal healing (Ventin-Holmberg et al., 2025). Although longer term longitudinal sampling, extending up to 2 years, was reported by Carlsen et al. (2024), mucosal microbiome and mucosal healing were not assessed. Future pediatric studies should standardize sampling to a fixed phase/day and integrate pharmacokinetic measures with microbiome models.

### 4.7 Concomitant medications and confounding factors

Corticosteroids, immunomodulators, and antibiotics were frequently reported but inconsistently controlled. These agents independently alter microbiome composition and represent significant confounding factors. Antibiotic exposure is particularly important, in Ventin-Holmberg et al. (2022), antibiotic use within 2 weeks before participation was associated with reduced *Faecalibacterium*, consistent with the known sensitivity of obligate anaerobes to antibiotic perturbation. Antibiotic exposure may therefore move a patient toward a non-responder microbial state even if inflammatory markers improve. On the other hand, several included studies directly tested whether concomitant medications independently drove the observed microbiome findings. Höyhtyä et al. (2023) confirmed that antibiotic and steroid-associated bacterial shifts were not significantly associated with IFX treatment response, Kolho et al. (2015) concluded that concomitant glucocorticoids and methotrexate did not exert a drastic influence on overall microbiota composition; and Carlsen et al. (2024) found no significant association between thiopurine or 5-ASA use and microbiome composition or diversity during IFX maintenance. These findings provide reassurance that the response-associated microbiome signatures described in this review mostly are IFX-related rather than unmeasured co-medication effects.

Additional sources of heterogeneity include small sample sizes, methodological variability (16S, shotgun sequencing, qPCR), disease subtype, and environmental influences such as diet and geographical location.

### 4.8 Implications for healthcare professionals

Current evidence suggests early IFX response in pediatric IBD should be judged primarily by established clinical and biochemical markers, particularly symptom improvement and fecal calprotectin, rather than by expecting uniform recovery in overall microbial diversity. According to the reviewed studies, clinical and biochemical outcomes improved consistently with response, whereas alpha diversity changes were heterogeneous and remission could persist despite limited microbiome recovery. Lack of broad diversity restoration should not be interpreted in isolation as treatment failure. Nevertheless, the microbiome may still have near-term translational value for stratification. Baseline enrichment of taxa such as *Faecalibacterium*, *Subdoligranulum*, and *Bifidobacterium*, and lower abundance of dysbiosis-linked organisms, were more consistently associated with favorable IFX response than diversity metrics alone. Therefore, where microbiome profiling is available in research settings, clinicians should interpret it as an adjunct to standard monitoring, with greater emphasis on compositional response signatures than on alpha diversity alone. However, routine microbiome-guided IFX decision-making cannot yet be recommended until larger pediatric studies validate reproducible, clinically usable signatures.

### 4.9 Strengths, limitations, and future directions

This review has several important strengths. To our knowledge, it represents the first systematic synthesis specifically evaluating IFX related microbiome changes in pediatric IBD. The analysis distinguished induction and maintenance phases, allowing clearer interpretation of temporal microbiome dynamics. Integrated taxonomic and functional outcomes, and incorporated responders vs non-responder comparisons aligned with the review objectives.

However, important limitations remain. First, despite a rigorous search across four databases, there always remains some risk that literature may not have been captured. Moreover, the review included 13 studies (Carlsen et al., 2024; Kowalska-Duplaga et al., 2020; Höyhtyä et al., 2023; Wang et al., 2021; Wang et al., 2017; Taylor et al., 2020; Stein et al., 2022; Salamon et al., 2020; Lv et al., 2024; Kowalska-Duplaga et al., 2019; Kolho et al., 2015; Hurych et al., 2024; Ventin-Holmberg et al., 2022), all of which were non-randomized observational studies, which limits causal inference. Therefore, the current evidence cannot determine whether microbiome composition directly influences IFX response or whether microbial changes mainly reflect reduced intestinal inflammation after treatment. The microbiome signatures identified in this review should be considered exploratory, associative biomarkers rather than validated mechanistic predictors of treatment response. Many of these studies had small cohorts, potentially limiting statistical power and increasing the risk of selection bias. Moreover, some studies (Kolho et al., 2015; Hurych et al., 2024) included in the review were composed of pediatric datasets which included anti-TNFα exposure without isolating IFX-specific effects or combining remission groups, potentially limiting the attribution to IFX. Finally, several studies originated from similar research settings and author groups, although we did not find explicit cohort overlap (specifically with reported outcomes), potential overlap could not be definitively ruled out and should be considered when interpreting the independence of the evidence base.

## 5 Conclusion

This systematic review suggests that, in pediatric IBD, IFX therapy is associated with selective compositional and functional shifts rather than broad restoration of microbial diversity. Across 13 studies, IFX improved clinical and biochemical outcomes in a consistent manner, but the microbial response was compositionally and functionally targeted, as we report enrichment of SCFA-producing commensals (*Faecalibacterium, Subdoligranulum, Bifidobacterium*) as well as reduction in dysbiosis-associated taxa (Gammaproteobacteria, *Candida*), rather than broad diversity restoration. We reported a dissociation between rapid pharmacodynamic suppression of inflammation and slower/partial microbiome recovery, implying that anti-TNFα efficacy and ecological repair follow distinct timescales. Three findings carry direct translational relevance: baseline microbiome composition may function as a candidate pharmacodynamic biomarker of response; with SCFA-producing taxa enriched in responders and Proteobacteria dominated dysbiosis tracking with non-response. Second is that the microbiome seems to interact with IFX pharmacokinetics as indicated in adult cohorts, but this remains unexamined in pediatrics and presents a clinical gap for future research. Third is that persistent dysbiosis, despite clinical remission, supports further investigation of microbiome-targeted adjuvant therapies to combine anti-TNFα treatment. These conclusions remain preliminary; we cannot yet recommend routine microbiome-guided IFX decisions. Advancing the field will require larger prospective pediatric cohorts, where longitudinal stool and mucosal samples are paired with IFX trough levels and ADA status, endoscopic outcomes and prespecified microbiome features, to enable integrated pharmacomicrobiomic models for precision biologics therapy in pediatric IBD.

## Data Availability

The original contributions presented in the study are included in the article/Supplementary Material, further inquiries can be directed to the corresponding author.
