# Supplementary material for: Infliximab-linked gut microbiome signatures as candidate treatment response biomarkers in pediatric inflammatory bowel disease: a systematic review
Source: Front Pharmacol. 2026 Jul 10;17:1877033. doi: 10.3389/fphar.2026.1877033 (PMC13395688; doi:10.3389/fphar.2026.1877033)
Supplement: Supplementary file 1 [file Table1.docx]

*PICO Framework with MeSH Terminology*

Table S1. PICO Framework Table with MeSH terminology.

| PICO | Description | Keywords | MeSH Terms |
| --- | --- | --- | --- |
| Population | Children and adolescents with inflammatory bowel disease (Crohn’s disease, ulcerative colitis) | pediatric, paediatric, child*, adolescen*, teen*, youth, inflammatory bowel disease, Crohn*, ulcerative colitis, IBD | Child; Adolescent; Inflammatory Bowel Diseases; Crohn Disease; Colitis, Ulcerative |
| Intervention | Infliximab | Infliximab, Remicade | Infliximab |
| Comparator | Pre- vs. post-treatment, baseline vs. treatment, responders vs. non-responders | baseline, pre-treatment, comparator, control, responder, non-responder, treatment outcome | \| Treatment Outcome \| \| --- \|  \|  \| \| --- \| |
| Outcomes | Gut microbiome: composition, diversity, function, associations with response | microbiome, microbiota, gut flora, dysbiosis, metagenomic, 16S, shotgun sequencing, alpha diversity, beta diversity | Gastrointestinal Microbiome; Microbiota |

* Indicates that word variations should be explored in the search strategy.
