# Supplementary material for: Infliximab-linked gut microbiome signatures as candidate treatment response biomarkers in pediatric inflammatory bowel disease: a systematic review
Source: Front Pharmacol. 2026 Jul 10;17:1877033. doi: 10.3389/fphar.2026.1877033 (PMC13395688; doi:10.3389/fphar.2026.1877033)
Supplement: Supplementary file 2 [file Table2.docx]

**Supplementary Table 2.** Terms and definitions used in this systematic review.

| **Term** | **Working definition for this review** | **Definition Reference** |
| --- | --- | --- |
| ***Clinical and Treatment Terms*** | | |
| **Inflammatory bowel disease (IBD)** | A chronic, relapsing group of immune-mediated inflammatory disorders of the gastrointestinal tract that includes Crohn's disease and ulcerative colitis; in pediatrics, classification may also include IBD-unclassified. | (34) |
| **Crohn's disease (CD)** | An IBD subtype that can affect any part of the gastrointestinal tract and is commonly characterized by patchy, transmural inflammation. | (34) |
| **Ulcerative colitis (UC)** | An IBD subtype limited to the colon, with continuous mucosal inflammation that usually begins in the rectum. | (34) |
| **IBD-unclassified (IBD-U)** | Chronic inflammatory bowel disease with colonic features of IBD but insufficient criteria to classify the case confidently as Crohn's disease or ulcerative colitis. | (34) |
| **Infliximab (IFX)** | A chimeric monoclonal antibody directed against tumor necrosis factor-alpha (TNF-α), used as a biologic therapy in inflammatory bowel disease. | (35) |
| **Anti-TNFα therapy** | Biologic treatment that reduces inflammation by neutralizing TNF-α signaling. | (36) |
| **Induction therapy** | The initial treatment phase intended to achieve remission; with infliximab this is commonly the early dosing phase at weeks 0, 2, and 6. | (37) |
| **Maintenance therapy** | Ongoing scheduled treatment administered after induction to sustain remission over time. | (37) |
| ***Microbiome and Assay Terms*** | | |
| **Gut microbiome** | The community of microorganisms in the gastrointestinal tract, together with their collective genetic material and functional potential. | (9) |
| **Dysbiosis** | An altered microbiota state involving changes in composition and diversity, with an imbalance between commensal and potentially pathogenic microorganisms. | (38) |
| **Alpha diversity** | Within-sample diversity reflecting richness, evenness, or both, within a single microbial community. | (39) |
| **Beta diversity** | Between-sample differences in microbial community composition. | (39) |
| **Taxonomic composition / abundance** | The identity and relative distribution of microbial taxa in a sample, such as phyla, genera, or species. | (39) |
| **16S rRNA gene sequencing** | A targeted marker-gene sequencing approach used mainly for bacterial taxonomic profiling by sequencing variable regions of the 16S ribosomal RNA gene. | (39) |
| **Shotgun metagenomic sequencing** | Untargeted sequencing of total DNA in a sample to profile both microbial taxa and functional gene content. | (39) |
| **Amplicon Sequence Variant (ASV) / Operational Taxonomic Unit (OTU)** | Amplicon sequence variants (ASVs) are exact sequence features resolved to single-nucleotide differences; operational taxonomic units (OTUs) are clusters of similar sequences grouped using a predefined similarity threshold. | (40) |
| **qPCR** | Quantitative polymerase chain reaction, used to detect and quantify a predefined nucleic-acid target in real time. | (41) |
| ***Outcome Terms*** | | |
| **Clinical remission / response** | Improvement to predefined disease-activity thresholds or meaningful clinical improvement, usually assessed with validated clinical, biochemical, and/or endoscopic measures. | (42) |
| **Fecal calprotectin (FC)** | A stool biomarker of intestinal neutrophil-driven inflammation that is widely used as a noninvasive marker of inflammatory bowel disease activity. | (43) |
| **Functional / metabolomic findings** | Predicted or measured microbial functions, pathways, or metabolites (for example bile acids or short-chain fatty acids) reported in addition to taxonomic composition. | (44) |
